# Supplementary material for: The Difference in the Proportions of Deleterious Variations within and between Populations Influences the Estimation of FST
Source: Genes (Basel). 2022 Jan 22;13(2):194. doi: 10.3390/genes13020194 (PMC8872184; doi:10.3390/genes13020194)
Supplement: Supplementary file 1 [file genes-13-00194-s001.zip › genes-1549590-supplementary.pdf]

Supplementary Information

Temporal distribution of deleterious variations influences the estimation of F_{ST}

Sankar Subramanian

GeneCology Research Centre, The University of the Sunshine Coast, 90 Sippy Downs Drive, Sippy Downs Qld 4556, Australia

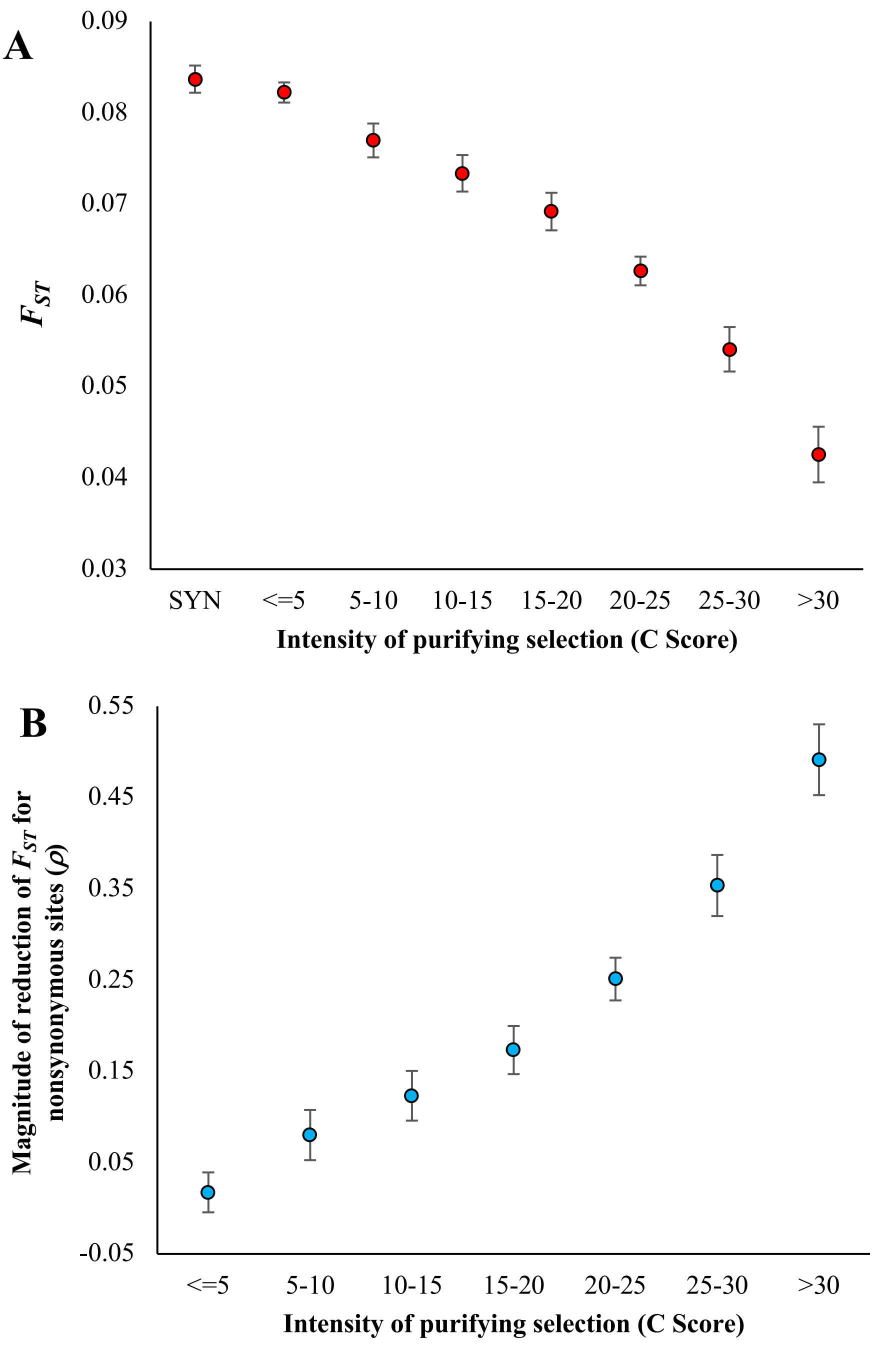

Figure S1. **(A)** Relationship between selection intensity and F_{ST} using Nei's estimator (G_{ST}). Whole exome data comprising synonymous SNPs (sSNPs) and nonsynonymous SNPs (nSNPs) for the Italian (TSI)-Nigerian (YRI) population pair was used to estimate F_{ST}. The magnitude of selection intensity on nSNPs is measured by the Combined Annotation-Dependent Depletion (CADD) method that integrates many diverse annotations into a single measure (*C* score). **(B)** Magnitude of reduction of F_{ST} estimates and selection intensity. X-axis shows the reduction in F_{ST} estimates of nSNPs in comparison with that of sSNPs (ρ) using equation 11 (see methods) for the exome data described above. Error bars show standard error of the mean.

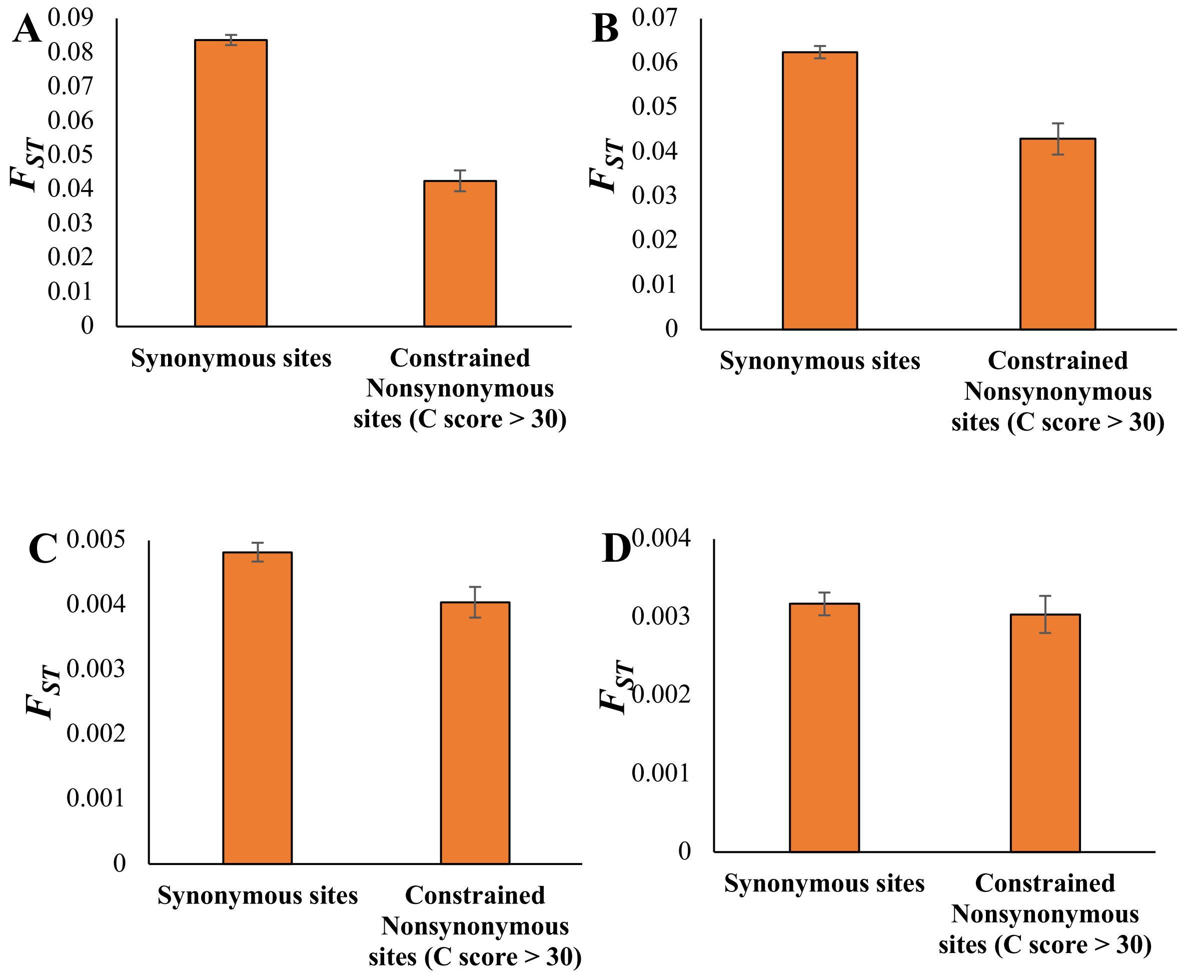

Figure S2. F_{ST} estimates (using Nei's estimator, G_{ST}) for synonymous and highly constrained nonsynonymous SNPs of the **(A)** Italian-Nigerian **(B)** Italian-Chinese **(C)** Italian-British and **(D)** Italian-Spanish population pairs. Error bars are the standard error of the mean. The difference between the FST estimates of neutral and constrained sites are highly significant ($P < 0.01$, Z test) for three comparisons and not significant for the Italian-Spanish pair.

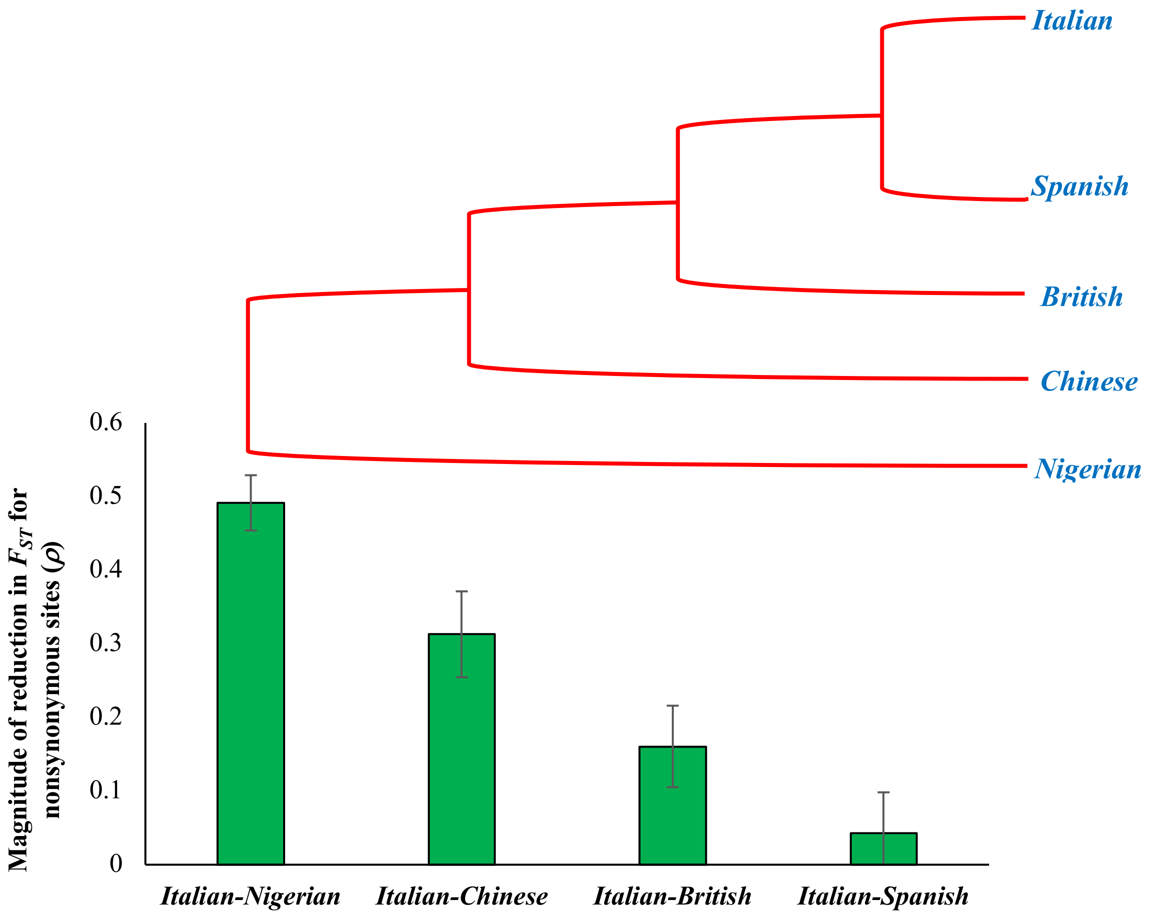

Figure S3. The magnitude of reduction in F_{ST} estimates (using Nei's estimator, G_{ST}) of nSNPs obtained for four population pairs. The population tree on top is drawn to highlight the correlation between the population divergence and the magnitude of reduction in F_{ST}.
